# Monitoring Kikuletwa river levels in northern Tanzania: A data set unlocking insights for effective flood early warning systems

**DOI:** 10.1016/j.dib.2023.109395

**Published:** 2023-07-12

**Authors:** Lawrence Mdegela, Yorick De Bock, Edith Luhanga, Judith Leo, Erik Mannens

**Affiliations:** a University of Antwerp – imec IDLab, Department of Computer Science Sint-Pietersvliet 7, 2000 Antwerp, Belgium; b The Nelson Mandela African Institution of Science and Technology, P.O Box 447, Arusha, Tanzania; c Carnegie Mellon African University, P.O Box 6150, Kigali, Rwanda

**Keywords:** River levels, Flood early warning, Data scarcity, Ungauged rivers, Sensors

## Abstract

Floods are a recurring natural disaster that pose significant risks to communities and infrastructure. The lack of reliable and accurate data on river systems in developing countries has hindered the development of effective flood early warning systems. This paper presents a data set collected using ultrasonic distance sensors installed at two locations along the Kikuletwa River in the Pangani River Basin, Northern Tanzania. The dataset consists of hourly measurements of river water levels, providing a high-resolution time series that can be used to study trends in water level changes and to develop more accurate flood early warning systems.

The Kikuletwa River dataset has significant potential applications for flood management, including the calibration and validation of hydrological models, the identification of critical thresholds for flood warning, and the evaluation of flood forecasting techniques. The dataset can also be used to study the hydrological processes in the basin, such as the relationship between rainfall and river discharge, and to develop more efficient and effective flood management strategies.

The ultrasonic distance sensors were configured to record river level data at hourly intervals, providing a continuous time series of river levels. The data was subjected to quality control procedures to ensure accuracy and consistency, and missing or erroneous data was corrected or removed where necessary.

**Specifications Table**

**Table:** 

Subject	Data Science
Specific subject area	Big Data Analytics
Type of data	Time series, Figures
How the data were acquired	Ultrasound sensors were used to collect data on the river level. These sensors were affixed to a Waspmote Plug & Sense SmartAgriculture PRO board, which was mounted on the bridge. The ultrasound sensor measured the distance between the sensor and the water surface. Using the initial river depth measurements obtained at each location, the subsequent river levels were calculated.
Data format	Raw Pre-processed
Description of data collection	A feasibility study was conducted on-site to determine optimal locations for sensor deployment along the Kikuletwa river. This study identified two suitable locations, namely the Kikavu bridge and the Kikuletwa bridge. Afterward, the sensors were physically installed, configured, and programmed. During the dry season, the initial river depth was measured at both locations, and the sensors began recording water level measurements on an hourly basis. The data generated by the sensor nodes are transmitted via a cloud bridge and forwarded to a central database for storage and visualization in their original form.
Data source location	Town: Moshi Region: Kilimanjaro Country: Tanzania Coordinates: The Kikavu bridge is situated at a latitude, longitude [-3.44, 37.30], representing the more upstream of the two locations while the Kikuletwa bridge is situated at a latitude, longitude [-3.55, 37.31] representing the more downstream of the two locations. Generally, The Kikuletwa catchment, situated in the north-western region of the Pangani River basin, encompasses a vast area of approximately 6650 km2 as detailed in [1]. It spans across multiple administrative districts, comprising a total of 80 administrative wards. The catchment is primarily characterized by its drainage system, consisting of 15 major rivers that originate from the renowned Mount Meru and Mount Kilimanjaro. These rivers converge to form the principal Kikuletwa River, which subsequently flows into the downstream Nyumba ya Mungu reservoir. This catchment plays a crucial role in the hydrological dynamics of the Pangani River basin, influencing water availability, runoff patterns, and overall ecosystem health. The intricate interplay between topography, climatic conditions, and land use within the catchment necessitates a comprehensive understanding of its hydrological processes for effective water resource management and flood mitigation strategies.
Data accessibility	Repository name: Zenodo Data identification number: doi.org/10.5281/zenodo.7890758 Target URL to the data: https://zenodo.org/badge/latestdoi/632186294
Related research article	Mdegela L, De Bock Y, Municio E, Luhanga E, Leo J, Mannens E. A Multi-Modal Wireless Sensor System for River Monitoring: A Case for Kikuletwa River Floods in Tanzania. *Sensors*. 2023; 23(8):4055. https://doi.org/10.3390/s23084055[2]

## Value of the Data

• The dataset provides valuable insights into the water level fluctuations of the Kikuletwa River in Northern Tanzania, allowing for better understanding of the river's hydrological behavior.
• Researchers, policymakers, and disaster management authorities can benefit from these data to develop more accurate flood early warning systems and improve disaster preparedness.
• The data can be used as a basis for hydrological modeling studies, which can contribute to the development of more effective flood management strategies in the region.
• Unprecedented data collection: This dataset stands out as the first comprehensive and long-term collection of water level data from the Kikuletwa River. Previous monitoring efforts were limited in their frequency and duration, providing only fragmented insights. The new dataset, collected hourly, offers an unprecedented level of detail, enabling researchers and stakeholders to capture fine-scale variations in water level fluctuations and better characterize the river's behavior.

## 1 Objective

The dataset presented in this paper addresses the challenge of data scarcity in underexplored river catchments in Northern Tanzania, specifically regarding river level monitoring. The use of ultrasonic distance sensors to collect and analyze river level data from the Kikuletwa River provides a high-resolution and continuous time series that is essential for the development of accurate flood early warning systems and improving disaster preparedness in the region. In addition, the dataset can be used to calibrate and validate hydrological models, identify critical thresholds for flood warning, and evaluate flood forecasting techniques, ultimately leading to more efficient and effective flood management strategies. The availability of high-quality, continuous data on river levels is also critical for improving the accuracy of machine learning models used in flood prediction [3]. With its hourly measurements, the Kikuletwa River dataset provides a rich source of data for training and testing machine learning algorithms, enabling the development of more robust models that can provide timely and accurate flood warnings.

## 2 Data Description

The article presents two raw data files in CSV format, which contain hourly river level measurements from two separate locations along the Kikuletwa River. Both files include data spanning from April 2022 to April 2023. The data files are:

i. Kikavu.csv: This file contains river level measurements from the first location where sensors are installed. The data consists of two columns: 'time' and 'river_level'. The 'time' column represents the timestamps of data collection, while the 'river_level' column contains the corresponding river level measurements in meters.
ii. Kikuletwa.csv: This file contains river level measurements from the second location where sensors are installed. Like the first file, it also has two columns: 'time' and 'river_level'. The 'time' column represents the timestamps of data collection, and the 'river_level' column contains the corresponding river level measurements in meters.

Both data files can be accessed at a repository found at [4]. The data is still actively collected in real time and will be updated semiannually. The updates will be provided in two forms. Firstly, improvements will be made to the quality of the existing data for the previous period. This includes refining data collection methods, enhancing data validation processes, and addressing any identified issues or inconsistencies. Secondly, additional data sets will be incorporated as they become available. This ensures that the dataset maintains both its quality and continuity over time, enabling a comprehensive and reliable source of data.

### 2.1 Data visualization

**Fig. 1:**
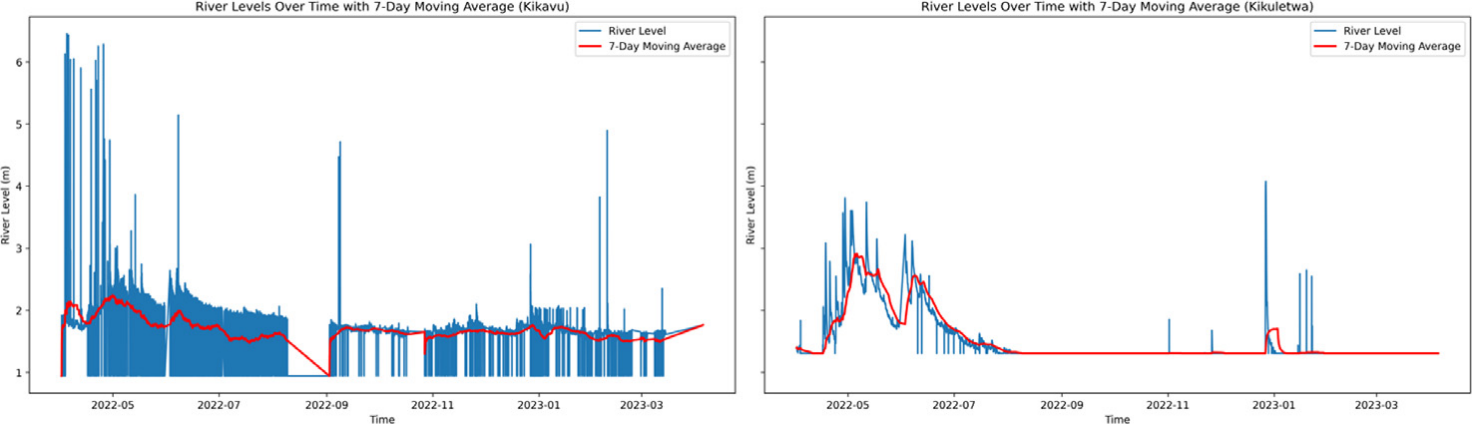
River levels over time with 7 day moving average from Kikavu bridge (left) and Kikuletwa bridge (right).

Multiple plots were created for each location to visualize the river level data. Line plots were used to show the evolution of the data over time for both locations, as seen in Fig. 1. By plotting the data points and connecting them with lines, line plots allow to observe trends, patterns, and any changes in the dataset's values. Additionally, the inclusion of a 7-day moving average in the line plot helps smoothen out short-term fluctuations and reveal underlying trends more clearly.

**Fig. 2:**
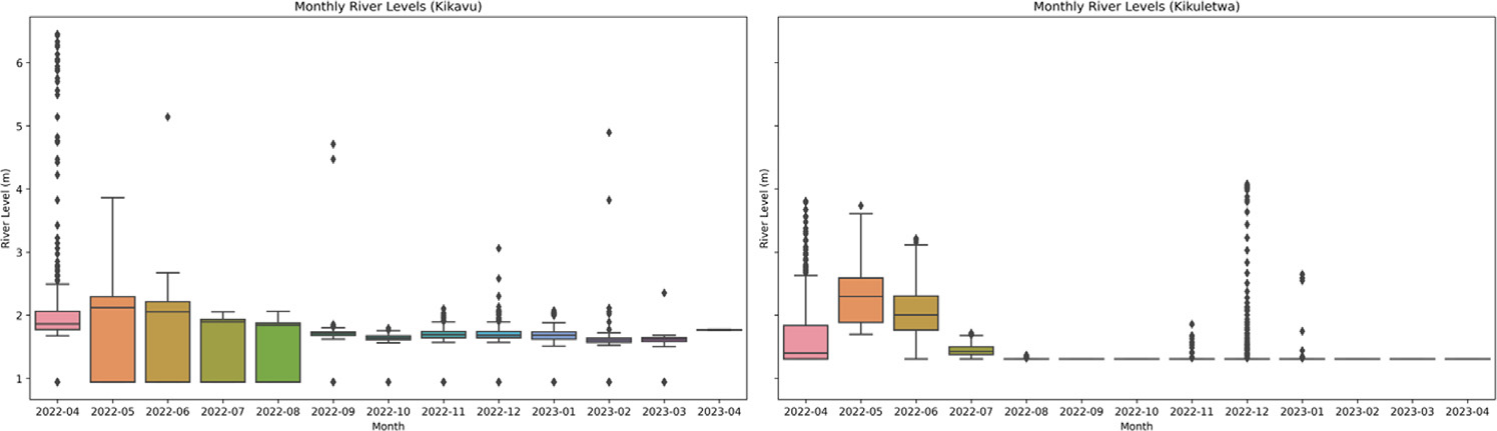
Box plots for monthly average river levels from sensor at Kikavu bridge (left) and Kikuletwa bridge(right).

To gain a deeper understanding of the data, box plots were plotted (Fig. 2) to showcase the range and distribution of the data. Box plots provide a visual summary of the data's statistical properties, including measures such as the minimum, maximum, median, and quartiles. They showcase the range and distribution of the data, highlighting any potential outliers or skewed distributions. Furthermore, box plots helped to understand the central tendency and variability of the dataset, enabling comparisons between different locations or time periods.

**Fig. 3:**
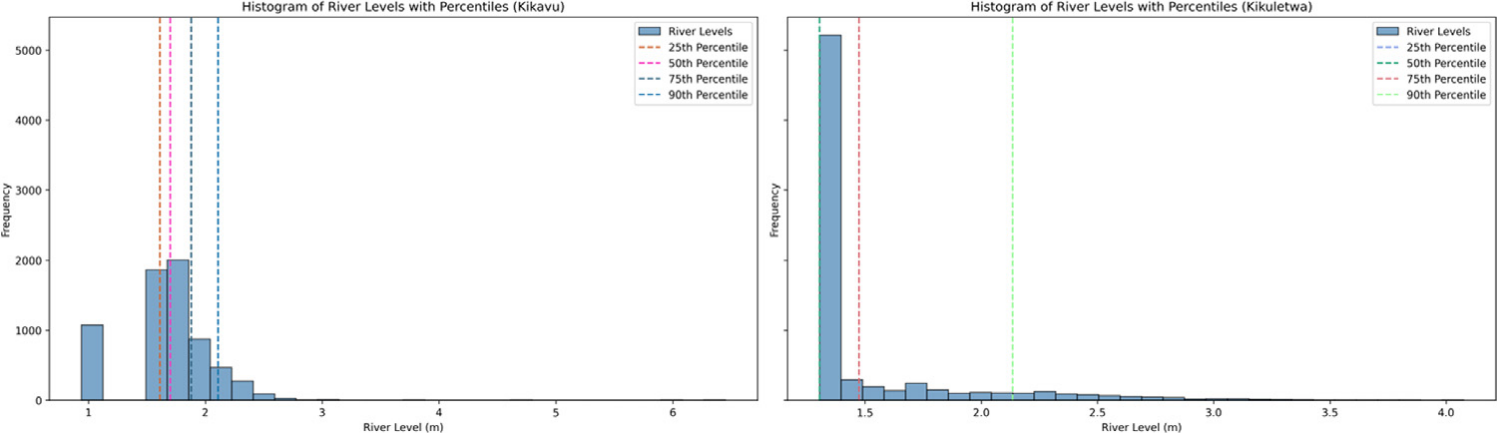
Histograms with percentiles for Kikavu bridge and Kikuletwa bridge.

Additionally, histograms were created (Fig. 3) to demonstrate the shape, central tendency, and variability of the dataset. By dividing the data into intervals or bins and displaying the frequency or proportion of observations within each bin, histograms allow to visualize the data's distributional characteristics. They also provide insights into the spread, and any clustering or gaps in its values.

**Fig. 4:**
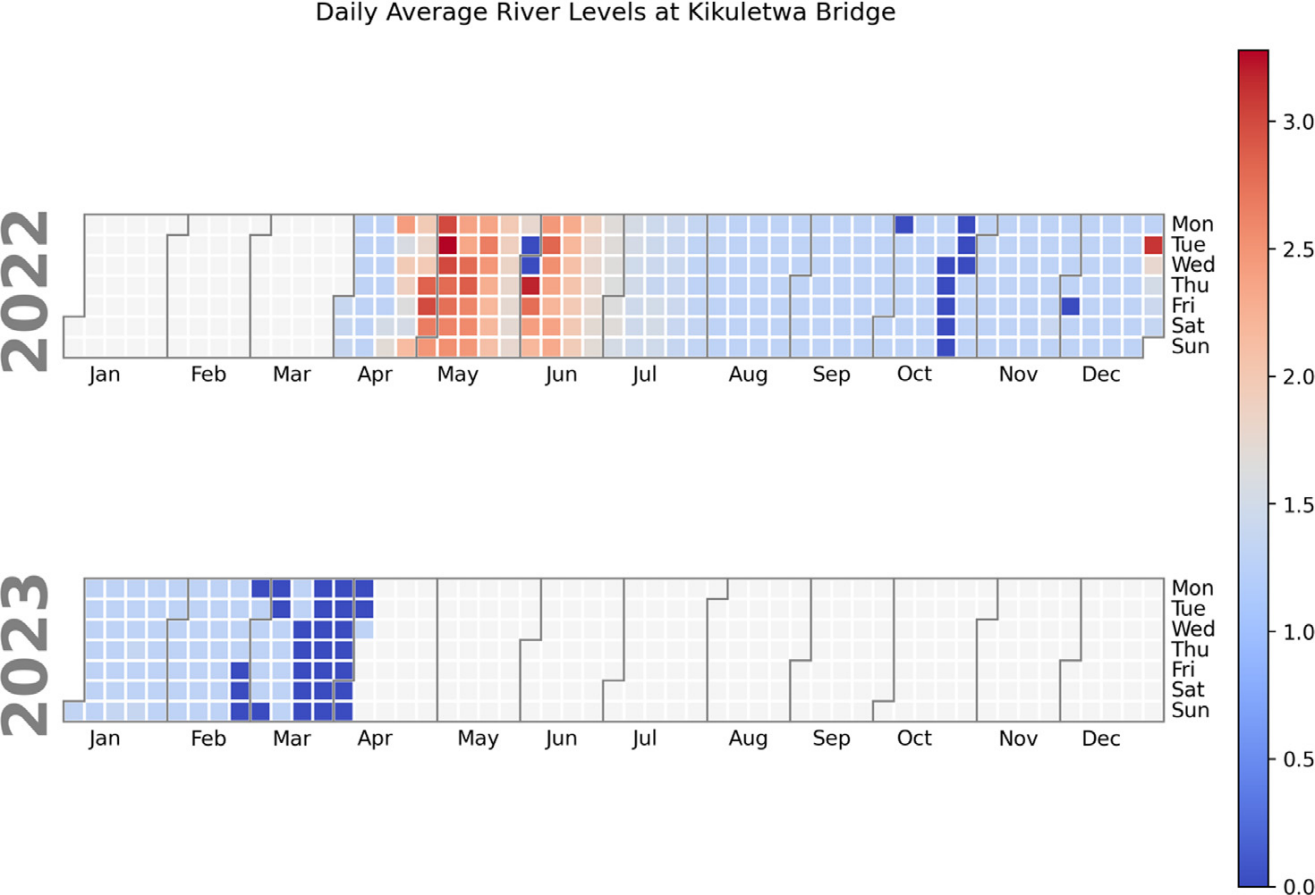
Calendar heatmap plot for daily river level averages from Kikuletwa bridge.

**Fig. 5:**
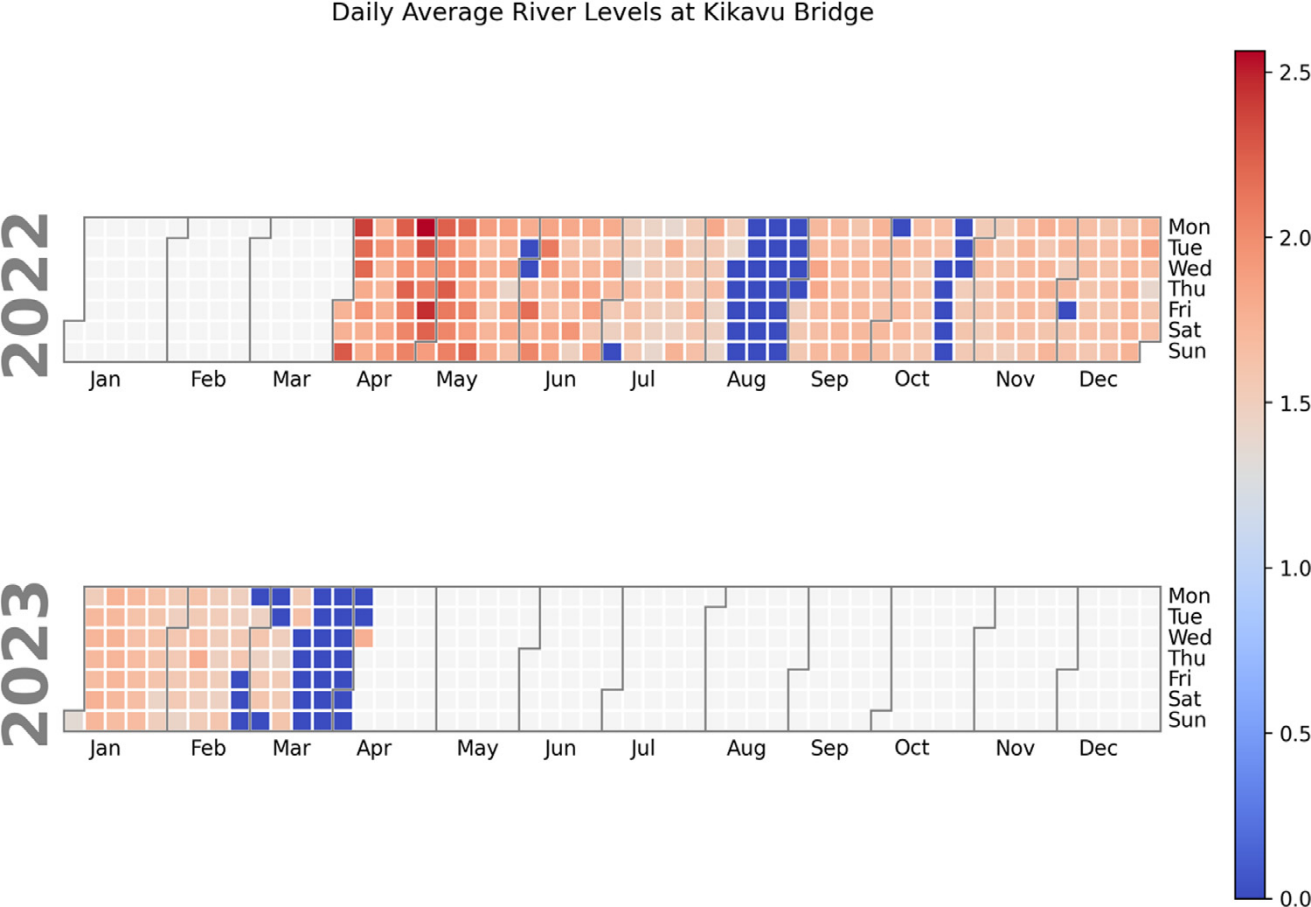
Calendar heatmap plot for daily river levels from Kikavu bridge.

To further explore additional insights into patterns and trends in the data that may not be easily visible in other types of plots we plotted calendar heatmaps (Fig. 4, Fig. 5). Calendar heatmaps were utilized to identify patterns and trends in the data across different days or periods. By representing the data on a calendar grid, heatmaps enable us to spot temporal patterns, such as seasonal variations or weekly trends. They facilitate the detection of outliers or anomalies occurring on specific days, enhancing our understanding of the data's temporal dynamics.

These different types of plots were selected to provide a comprehensive analysis of the dataset from various angles. Together, they offer valuable insights into the data's temporal behavior, statistical properties, distributional characteristics, and temporal patterns.

### 2.2 Missing data

There are a few instances of missing data, which can be attributed to three main factors. Firstly, occasional gaps occur at the beginning of each month due to untimely data bundle recharging, as the data bundles are renewed monthly. Secondly, updates to the cloud bridge infrastructure can sometimes disrupt the flow of data, causing intermittent interruptions. These occurrences are unpredictable. Thirdly, there may be gaps in the data caused by sporadic sensor malfunctions. However, it is important to note that none of these issues have significantly affected the overall quality of the collected data.

The refined data collection frequency helps mitigate some of the data gaps, as the river levels do not exhibit sudden and drastic changes within each period. This allows for better management and analysis of the available data, despite occasional gaps.

Efforts are continuously being made to address these challenges and ensure the data collection process is as robust and reliable as possible.

## 3 Experimental Design, Materials and Methods

### 3.1 The board and the sensors

**Fig. 6:**
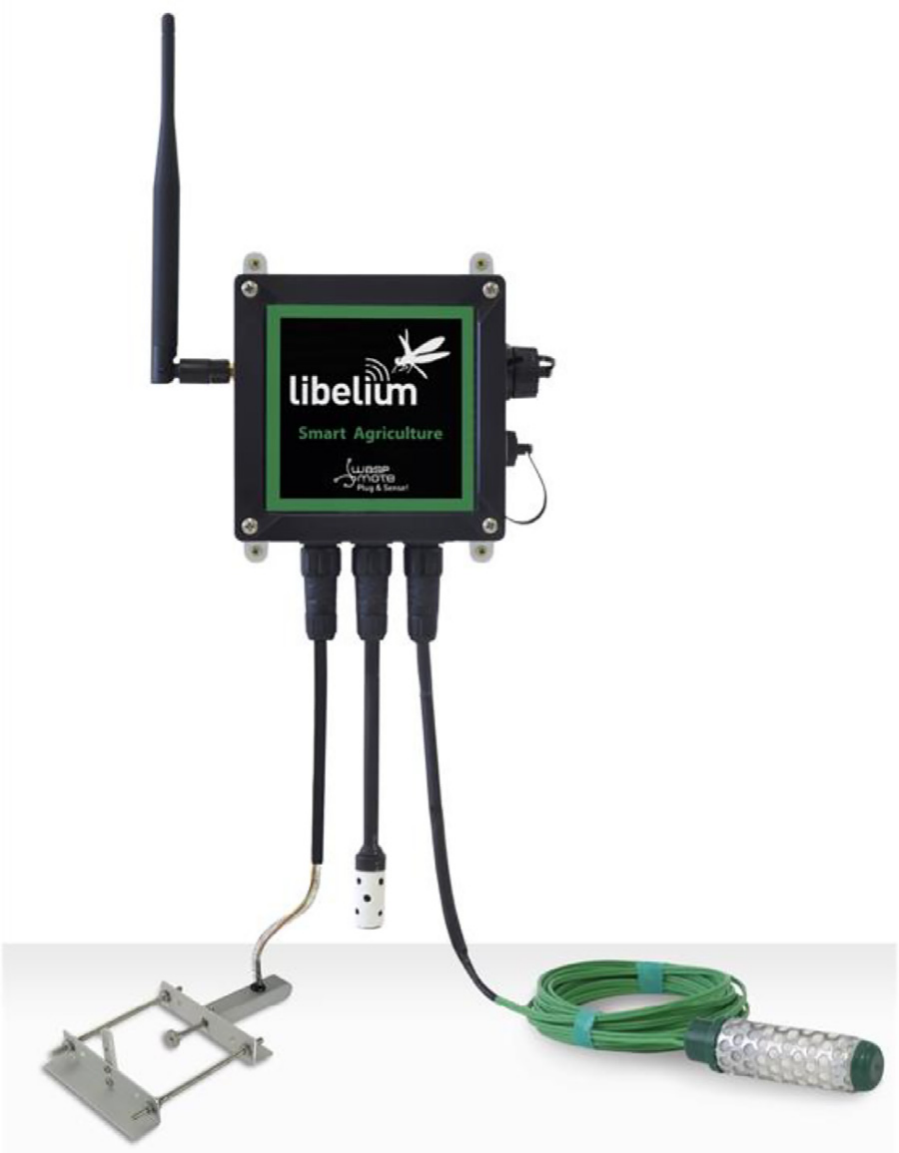
Waspmote Plug & Sense! Smart Agriculture PROSmart Agriculture PRO.

The I2CXL-MaxSonar®-MB7040™ attached to a Libelium Waspmote Plug & Sense Agriculture PRO v30 (Fig. 6) [5], a high-performance ultrasonic sensor created by MaxBotix™, is specifically engineered for a diverse array of applications. With an operational frequency of 42 kHz, this sensor is capable of detecting objects at a maximum distance of 765 cm. The digital bus interface facilitates streamlined connectivity and communication with other devices.

The power supply for this sensor varies between 3.3 and 5 volts, offering adaptability for different systems. Concerning power consumption, the sensor necessitates an average current of 2.1 mA at 3.3 V and 3.2 mA at 5 V. During peak performance, consumption may escalate to 50 mA at 3.3 V and 100 mA at 5 V. This efficient utilization of power renders it suitable for an extensive range of applications. A notable characteristic of the I2CXL-MaxSonar®-MB7040™ is its capacity to operate both indoors and outdoors, attributable to its IP-67 rated casing. This resilient design allows the sensor to endure harsh environmental conditions, making it an optimal choice for outdoor applications such as monitoring remote river levels.

Waspmote Plug & Sense Agriculture PRO employ a digital bus interface for data transmission during the measurement process and it is equipped with a 4G radio module to ensure seamless wireless communication, enabling high-speed connectivity to the LTE, HSPA+, and WCDMA cellular networks. It is optimized to work with internet servers, utilizing multiple application layer protocols internally to ensure smooth data transmission to the cloud. Furthermore, the device supports HTTP navigation and allows for the uploading and downloading of various contents to web servers. It also provides secure connections using SSL certificates and enables the configuration of TCP/IP private sockets. The File Transfer Protocol (FTP) is also supported, facilitating efficient file handling within an application.

On the power side, the devices comprise two kinds of batteries: a rechargeable lithium-ion battery with a capacity of 6600 mAh and a nominal voltage of 3.7 V, and a non-rechargeable battery with a nominal voltage of 3.4 V and a capacity of 52,000 mAh. To ensure appropriate battery charging current, Waspmote has a control and safety circuit in place. Besides, these devices are furnished with a solar panel that serves as a power source. The Waspmote device features a stiff solar panel, capable of generating a maximum of 7 V and 500 mA, permitting a maximum charging current of 300 mA and up to 12 V.

Further configurations were done on the sensor nodes as follows:

• Communication and the protocol of the destination block; in this case, 4G was set as a communication module.
• Sleep time, set at 3600 s (1 h) for energy efficiency. This is the amount of time the device spends in sleep mode before a new cycle (sensor reading + transmission is performed).
• Critical battery warning setting: three thresholds (60%, 40%, and 20%) were set. A warning packet is sent upon reaching each threshold.

### 3.2 Sensor setup and river depth measurement

Two locations were identified as optimal for installing sensors: Kikavu bridge and Kikuletwa bridge. Initial river levels at both locations were measured using bathymetry mechanism called echo sounding. The ultrasonic sensor at the Kikavu bridge was mounted at a height of 765 cm from the water surface, which coincided with normal flow, specifically during the dry season. To establish an initial reference point for the sensor, we measured the river depth at Kikavu and obtained a value of 94 cm. The same procedure was replicated at Kikuletwa bridge with the initial depth of 130.5 cm and the sensor mounted at a height of 400 cm from the water surface.

### 3.3 Data Acquisition

Real-time data gathered by the sensor nodes is transmitted through the cloud bridge to the cloud service. To enable effective data management and analysis, the cloud service leverages a comprehensive dashboard. Microsoft Azure, a sophisticated cloud computing platform, serves as the foundation for the cloud service. To handle the influx of data streams from the sensor nodes, a virtual machine with the necessary capabilities is utilized. InfluxDB [6], a high-performing time-series database created to manage large amounts of time-stamped data, is used to manage, and analyze the data effectively. Additionally, Grafana [7], a powerful open-source visualization platform, is utilized to facilitate real-time data visualization, querying, and analysis. On the virtual machine, a custom Python script was developed to ensure seamless data integration and processing. This script regularly checks for new data files every two hours and incorporates them into InfluxDB using InfluxDB's APIs for automatic and efficient data ingestion. To manage data storage requirements, data retention policies can also be configured using this script.

### 3.4 Data Preprocessing

The raw data consisted of ultrasound distance (du) in centimeters from the sensor nodes readings. Having the initial river depth (di), means the total distance from the sensor node to the riverbed (dt) at respective sensor location was calculate as:

$$d_{t}=d_{u}+d_{i}$$

which means river levels at kikavu bridge (dkkv) were calculated as:which means river levels at kikavu bridge (dkkv) were calculated as:

$$d_{\text{kkv}}=d_{t}-d_{i}$$

the same procedure was used to calculate river levels at Kikuletwa bridge (d_kku_). Measurement in meters was preferable and so conversions from centimeters to meters were done.

Furthermore, the data was cleaned, where we removed incorrect, incomplete, and irrelevant data, such as duplicate entries, missing values, and outliers. Specifically, NumPy library was used to handle the multi-dimensional arrays that the data was stored in, as well as perform functions such as indexing, slicing, reshaping, and sorting of the data. Pandas library to handle data manipulation and analysis for read and write the data, handle missing values, group and aggregate data, merge and join data sets.

For handling outliers and scaling of the data, we utilized the Scikit-learn library which offers a variety of functions for data pre-processing, such as scaling, normalization, and feature extraction.

Finally, Matplotlib and Seaborn were used for data visualization to create the graphs, charts, and other visualizations that helped with the exploration and analysis of the data.

## Ethics Statements

The work presented in this paper meets all the ethical requirements for publication in Data in Brief. There isn't any part of the work that involved human subjects, animal experiments, neither does any data was collected from social media platforms.

## Declaration of Generative AI and AI-Assisted Technologies in the Writing Process

During the preparation of this work the author(s) used OpenAI-ChatGPT to improve language and readability. After using this tool/service, the author(s) reviewed and edited the content as needed and take(s) full responsibility for the content of the publication.

## CRediT authorship contribution statement

**Lawrence Mdegela:** Conceptualization, Methodology, Software, Formal analysis, Visualization, Writing – original draft. **Yorick De Bock:** Data curation, Methodology, Software, Writing – review & editing. **Edith Luhanga:** Supervision, Project administration, Writing – review & editing. **Judith Leo:** Supervision, Project administration. **Erik Mannens:** Supervision, Writing – review & editing.

## Declaration of Competing Interest

The authors declare that they have no known competing financial interests or personal relationships that could have appeared to influence the work reported in this paper.

## Data Availability

KikuletwaData (Original data) (LawrenceMdegela/KikuletwaData).
